# The Interpretation of Carbon Nanotubes’ Electrochemistry: Electrocatalysis and Mass Transport Regime in the Apparent Promotion of Electron Transfer

**DOI:** 10.3390/bios15020089

**Published:** 2025-02-05

**Authors:** Josipa Dugeč, Ivana Škugor Rončević, Nives Vladislavić, Josip Radić, Maša Buljac, Marijo Buzuk

**Affiliations:** 1 Department of General and Inorganic Chemistry, Faculty of Chemistry and Technology, University of Split, 21000 Split, Croatia; josipa.dugec@ktf-split.hr (J.D.); skugor@ktf-split.hr (I.Š.R.); nives@ktf-split.hr (N.V.); 2 Department of Analytical and Environmental Chemistry, Faculty of Chemistry and Technology, University of Split, 21000 Split, Croatia; jradic@ktf-split.hr (J.R.); masa@ktf-split.hr (M.B.)

**Keywords:** voltammetry, chronoamperometry, electrochemical impedance spectroscopy, (bio)sensors, carbon nanotubes, porosity, electrocatalysis, thin layer, diffusion

## Abstract

Carbon nanotubes (CNTs) are widely used in electrochemical sensors due to their significant impact on the electroanalytical signal. However, there remain significant doubts regarding the origin of the improved electroanalytical response observed in CNT-based sensors, particularly concerning the precise role of CNTs in these systems. In particular, the origin of the electrochemical response is controversial, as it may be due to either electrocatalytic or non-electrocatalytic processes. The latter implies that the electroanalytical response is mainly governed by the mass transport phenomena within the porous CNT layer. This article briefly reviews the several comprehensive models based on the role of porosity (diffusion in a ‘thin-layer’) on the electrochemical behavior as well as on the electrocatalytic properties of CNTs to resolve conflicts arising from misinterpretations of the electroanalytical response of CNT-based sensors. However, even though there are some explanations and conclusions on this topic, they seem to be valuable for specific electroactive species, the type of CNTs and/or electrode architecture, the electrode surface, etc. Accordingly, general theories and conclusions are not yet defined, so different approaches to this topic are still needed, since the main phenomenological effects responsible for the nature of the electrochemical response of the electrodes modified with CNTs need to be determined in a rational way.

## 1. Introduction

The modified electrodes have been broadly applied in the development of electrochemical sensors and biosensors [1]. By simply entering the keyword ((bio)sensor, carbon, nanomaterial, nanotubes, graphene, yarn, etc.) in the Web of Knowledge, a large number of published articles from the last 5 years—about 10,419—is listed.

As an attractive material, CNTs have many benefits that make them suitable for the development of electrochemical (bio)sensors, due to their apparent ability in the promotion of heterogeneous electron transfer (i.e., to act as an ‘electrocatalyst’) [2]. Here, we have to specify that the term ‘electrocatalytic’ refers to an improved electroanalytical response (e.g., lower overpotential and higher current signal) in the case of the electrode modified with a CNT layer in comparison to an unmodified one. However, there are disagreements concerning the origin of the enhanced electrochemical activity of CNTs. For example, direct electron transfer between carbon nanotubes and electrochemically active species has been reported in the literature [3,4], leading to the simple explanation that the enhanced electrochemical response of the electrode modified with CNTs is the consequence of the highly specific electroactive surface area. Although pristine CNTs may show inherent electrochemical activity, the most notable explanation is that the active sites (prepared by oxidation) present on CNTs (hydroxyl, alcoholic, carbonyl, and carboxyl) are responsible for the electrochemical activity. In certain cases, for example, in sensing H_2_O_2_ with MWCNTs, the origin of the improved electrochemical signal is attributed to the presence of the residual metal impurities [5,6]. We can define all the above aspects with the ‘kinetic’ approach to elucidate the role of CNTs.

However, as the modification of the electrode by these materials usually involves the formation of multiple porous layers on the electrode surface [1], another approach can be used in elucidating the improved electrochemical response of the CNT-modified electrode. Basically, this means that the effects and experimental results can be analyzed in the light of mass transport in the pockets of solution present in the CNTs’ porous layer. This means that the ‘electrocatalytic’ behavior of the pristine CNTs can be alternatively explained by the mass transport phenomena in the CNTs’ porous layer for kinetic independent reaction.

Nevertheless, the boundary between the two approaches mentioned above is not sharp, as the phenomena of mass transport (Fick’s laws) and the kinetics of heterogeneous electron transfer (HET) (Butler–Volmer kinetics) must be taken into account in both. As the ‘kinetic’ approach uses semi-infinite diffusion (Fick’s law, Cottrell) in explanation of the electrochemical response; in the case when the response is mainly controlled by mass transport in the porous CNTs, the finite diffusion model is useful. The latter is often interrelated with the ‘thin-layer’ diffusion of the electroactive species that occurs in the porous CNTs. Despite this fact, ‘thin-layer’ diffusion is a phenomenon that is reserved for situations when mass transfer equilibrium is achieved rapidly, as electroactive species have immediate access to the electroactive sites of the electrode. This roughly means that the electroactive species are located within 0.001 cm of the electrode (working and auxiliary) surfaces (it should be noted that the thickness of the diffusion layer can even reach 500 μm [7]). In this case, this phenomena can be simply described by Faraday’s and Nernst’s laws [8].

Furthermore, the situation becomes complicated when electroactive species are present in the porous layer of the electrode and in the bulk of the electrolyte. An even more complicated situation can be expected when the electrochemical reversible reaction or even the slow kinetics of HET occur. Also, modified electrodes may exhibit a complex mass transport regime that is a combination of semi-infinite and ‘thin-layer’ diffusions, especially when the porous layer is electrochemically active, but the substrate (the electrode) is not [2]. In addition, many of the proposed explanations show variations depending on the electroactive species as well as the type of CNTs. For example, the electrochemical behavior of Fe(CN)_6_^4−^ on the SWCNT-modified glassy carbon electrode (GCE) or H_2_O_2_ on MWCNTs can be explained with the ‘thin-layer’ model, while this model shows many weaknesses in the case of etoposide and Fe(CN)_6_^4−^ on MWCNTs. Further problems can arise from the fact that a heterogeneous electrochemical process takes place in the porous layer, which can lead to an uneven distribution of the electrolyte and the electroactive species, which affects the kinetics of HET [4].

In summary, many details regarding the electrochemical properties of CNTs are still unclear and controversial and need to be clarified to gain a complete insight into the electrochemical processes occurring in these electrode systems.

The aim of this article is to provide an overview of the approaches used to elucidate the processes and phenomena that govern the electrochemical response of the electrode modified within a porous carbon layer. This means a comprehensive insight into theoretical models and simulations, not only specified for CNTs, but for general porous electrode systems: a conductive or non-conductive porous layer in combination with a conductive or non-conductive substrate. Although, at first glance, these models seem unrelated to CNTs, they can provide important insights that can be helpful in the assessment of the electrochemical properties of CNTs. In addition to this ‘non-electrocatalytic’ approach, here, we present an overview of recent efforts to explore the ‘electrocatalytic’ properties of CNTs.

## 2. Carbon Nanomaterials as ‘Workhorse’ in Electroanalysis

During the last 20 years, among all carbon nanomaterials, different forms of carbon nanotubes and graphene have emerged as the most promising materials in various electrochemical systems, especially in the various architecture of electrochemical sensors. The benefits of these materials as a ‘workhorse’ in the construction of electrochemical (bio)sensors arise from the fact that (a) they can act as a transducer; (b) they can serve as a support for a recognition element or/and mediator; and (c) they can act as a mediator themselves [9,10,11,12,13,14,15].

Despite the avalanche number of articles on the development of the various types of electrochemical (bio)sensors, there is still no complete understanding of the underlying physical and chemical phenomena that occur in the (bio)sensors based on different CNTs. This can be manifested in various explanations of the mechanism of the analyte recognition, even for very similar systems [16].

Accordingly, many electrochemists fall into the snare and usually explain the improved electrochemical behavior with these electrodes by its ‘inherent’ electric properties, while neglecting other phenomena. The latter arise from the morphology of these materials. Basically, carbon nanomaterials with a hollow structure have a large specific surface area and porosity, which contribute to the improved possibilities of electrochemical energy storage and conversion (supercapacitors and batteries). As CNTs have diameters on a nanoscale, the unexpected behavior of fluids and ions inside the CNT channel can be noticed, including enhanced ionic currents [17,18,19,20,21]. This can be applied in the development of the field-effect ion-transport devices [22,23,24]. Also, 2D carbon nanomaterials (such as graphene) show potential as useful electrode materials in the above context, due to possibility to be arranged in a layered structure. The above properties (porosity and layered structure) can be harnessed in the modification of these materials with various inorganic, organic, biological, and other recognition elements for (electro)chemical detection. This versatility is one of the main reasons why these materials are widely used in the development of (bio)sensors.

## 3. Origin of the Electrochemical Response

The origin of the electrochemical response of the pristine CNTs have occupied the attention of the many scientists and led to the publication of several papers with provocative titles [4,25,26,27,28,29,30]. As already mentioned, ‘pristine’ CNTs can exhibit ’electrocatalytic’ behavior due to the presence of metallic impurities [5,6]. Procedures (chemical or electrochemical) for the purification of CNTs in order to obtain ’really pristine’ materials involve exposure to the various oxidation agents, which can result in cutting of the CNTs’ ends followed by the formation of the various morphology defects and oxygen functional group on the edges. Furthermore, the formation of the oxygen-containing functional group can also occur on the sidewall (basal side) of the CNTs. As a consequence, these changes have a significant influence on the Fermi state of the electrons in the CNTs; as the oxygen-containing functional groups increase, the gentle trend of decline in conductivity can be noticed [31,32,33,34,35,36,37,38]. In particular, these functional groups can increase the (pseudo)capacity of the carbon materials via the Faraday reaction. However, the most important property of the formed oxygen-containing functional groups is their ability to act as a highly efficient active center for heterogeneous electron transfer with electroactive species. This implies their inherent electrocatalytic behavior. For example, for hydrogen peroxide and ascorbic acid, improved electrochemical signals for oxidized SWCNTs were observed [39].

In many cases, it is difficult to separate the influences of the various parts of the ‘pristine’ CNTs from the overall electrochemical behavior. In general, the origin of the electroactive behavior of CNTs has been studied in detail by the Compton group [40,41,42]. By comparison of the electrochemical behavior of the CNTs with the behavior of the basal and edge plane pyrolytic graphite, they concluded that the sidewalls of the CNTs are electrochemically inactive, in contrast to the edge of the CNTs or its defected parts. On the contrary, other studies demonstrated that the sidewall (basal) of the CNTs is electrochemically active, and that this signal will dominate in the overall electrochemical response [43,44].

## 4. The Roots of Thin-Layer Diffusion: How the Concept Emerged

Due to the strong van der Waals forces (π-π attraction), CNTs have a tendency to agglomerate and form bundles, which presents a huge problem for its application in sensor construction. In order to prevail this problem, numerous dispersion strategies for CNTs have been presented [45,46,47,48,49,50,51] or reviewed, as well as various methods for their ordered immobilization, such as covalent, non-covalent, or direct growth on different transducers (e.g., solid electrodes) [52,53,54,55,56]. However, covalent immobilization can lead to a deterioration of the intrinsic conductive properties of the CNTs, as the hybridization of the carbon atoms changes from sp^2^ to sp^3^. In the non-covalent immobilization, which is based on the van der Waals attraction between the surface of the electrode and the CNTs, the structure may collapse due to the weak interaction between them. Whatever, due to the simplicity, the most abundant method of modification with CNTs has been casting the dispersion of the CNTs (or their composite) onto the electrode surface. This causes the random physical distribution of the CNTs at the electrode surface with the non-uniform thickness of the prepared layer. The random thickness, porous morphology, and tendency to form bundles cause the CNT layer to act as a ‘sponge’ for electroactive species and also as a diffusion barrier for electroactive species present in the bulk. Also, these properties exclude the possibility of overlapping the diffusion layers on the surface of the CNTs placed on the electrode surface, which is necessary to achieve semi-infinite diffusion. Finally, this leads to a complex mass transport regime, which affects the overall electrochemical behavior of the CNT-modified electrode and has usually been misinterpreted as the ‘electrocatalytic’ property of the CNTs. As the condition in the porous structure of the CNT layer resembles those that can be simulated in the thin-layer cells, some of the electrochemical phenomena, such as peak-to-peak separation or the lower bias of the electrochemical reaction, can be interpreted by the relative diffusion in a finite ‘thin layer’.

The first report on ‘thin-layer’ electrochemistry was published during the 1960s [57,58,59,60,61] and, during the next 10 years of significant progress in this field of electrochemistry, was achieved, especially through the practical and theoretical works of the Hubbard group [8,62,63,64,65,66] and Tyrin [67]. This concept was developed for situations where the spatial distribution of the electroactive species was confined between electrodes with narrow gap. The advantages of this concept arise from the fact that mass transport can be practically neglected, as the equilibrium state (or more precisely, the finite diffusion conditions) is achieved very rapidly. Hence, the mathematical description and equations that describe thin-layer phenomena are greatly simplified and can be derived from Faraday’s law and the Nernst’s equation or even in the frameworks of the Butler–Volmer kinetics.

## 5. Modeling of the Mass Transport to and Within Porous Electrodes

Prompted by the fact that many authors observed that electrodes modified with carbon nanotubes exhibited faster electrode kinetics, the Compton group(s) set out to meticulously and critically research this phenomenon, resulting in several published articles from the late 2000s until now [41,68,69,70]. These articles included both experimental and simulated results to clarify the improvement of the electrochemical response for the carbon nanotube-modified electrodes.

The early work of the Compton group [68] on this subject was conceived by the comparison of a simulation (using the Butler–Volmer model coupled to Fickian diffusion) with experimental data obtained by the cyclic voltammetry of the ferrocyanide on a glassy carbon electrode modified with single-walled carbon nanotubes (SWCNTs). The authors used two mass transport models for the interpretation of the results: semi-infinite planar diffusion and a thin-layer diffusion model. The thin-layer model was used, because it reflects the large surface area. However, the authors did not mention whether this surface is electroactive or not. This study was performed by cyclic voltammetry, and as criterion peak-to-peak separation was used. The results obtained lead to the conclusion that the decrease in the peak-to-peak separation on the SWCNT-modified GCE “could arise due to electrocatalysis, a change in diffusional regime, or a combination of these two effects”. However, the authors were cautious in drawing conclusions about the electrocatalytic behavior of the CNTs when comparing the electrochemical response of the pristine GCE and the GCE modified with SWCNTs.

Another study on the oxidation of the more ‘practical’ analytes (dopamine and epinephrine) on SWCNT-modified GCE was reported by Keeley and Lyons [71]. In this report, they expose some flawed arguments used for assigning electrocatalytic properties to SWCNTs (or nanotubes in general). Using the Nicholson model [72] to interpret the differences in the electrochemical response obtained by CVs for the SWCNTs dispersed in dimethylformamide (DMF) and N-methyl-2-pyrrolidone (NMP), they concluded that the heterogeneous rate constant (k_0_) is larger when DMF is used as the dispersant. However, relying on the previously published reports [68], they were prudent in interpreting the experimental data, as they were aware that the voltammetric behavior reflects processes that can be interpreted as a combination of semi-infinite and thin-layer diffusion. They demonstrated that the electrochemical response has a contribution from at least one ‘parasitic’ process that is a consequence of the adsorption of the dopamine onto SWCNT surface or its presence within the porous SWCNT layer. Since the results of the peak-to-peak separation indicate that no significant contribution from thin-layer effects is expected at high scan rates, they assume “that the reaction occurs not only at the outer surface of the nanotubes but also at reactive sites within the adsorbed assembly”. In addition, they go one step further by using potential step chronoamperometry to gain further insight into the phenomena. As was expected, the obtained results drastically deviated from the semi-infinite Cottrell model at longer times. Based on the above, they concluded that the scan rates have a significant influence on the ‘active surface’ of the modified electrode. As indicated, the authors were not explicit concerning the electroactivity of the nanotubes, but implicitly, it seems that they consider porous layer to be conductive.

Further evidence of the thin-layer phenomena was observed in the study of the irreversible oxidation of nicotine onto multi-walled carbon nanotube (MWCNT)-modified basal plane pyrolytic graphite (MWCNT-BPPG) [2]. In this investigation, the influence of the layer thickness on the current response was also studied. The authors showed that the peak current is almost linear with the amount of the MWCNT. Also, a ‘pre-adsorption’ effect on the current peak was observed “due to the delayed exchange of solution in the ‘thin layer’ within the MWCNT layer and the bulk solution”. According to the authors, the calculated slope of 0.62 of the log i vs. log υ dependence was attributed to the possible mixed mass transport regime comprised of the thin-layer diffusion in MWCNTs and semi-infinite diffusion in the bulk. The contribution of the thin-layer diffusion was higher for a thicker MWCNT layer, as the slope obtained was 0.78. As a consequence of the rapid depletion of the MWCNT layer by nicotine, the cyclic voltammogram deviates significantly from what is expected for semi-infinite diffusion. For the further illustration of the contribution of the thin-layer phenomena, cyclic voltammetry simulations at various scan rates were performed. As a model, vertically oriented cylindrical electrodes, of which only the sides are considered conductive, on an insulating substrate was used. Although this model is far from the real situation where the MWCNTs are randomly oriented, the essence of the phenomena is retained. The simulation shows that the slope of log i vs. log σ (where σ is the normalized scan rate) varies from 0.5 at the lowest σ, since at this scan rate, the size of the thin layer becomes negligible compared to the diffuse layer, towards 1 at intermediate scan rates, where the situation is reversed. Finally, at high scan rates, a slope of 0.5 is reached again. From this, the authors conclude that the thin-layer effects depend on the relationship between the size of the diffuse layer and the separation of the cylinders as well as their height.

A simulation study on the influence of the mass transport regime on the electrochemical response obtained by linear sweep and various pulse voltammetry was reported by Laborda et al. [1]. First, the change in the current signal was analyzed by altering the kinetic parameter of the electrode (from a completely irreversible to a reversible process) and the pulse height for the semi-infinite diffusion model. Subsequently, the dependence of the shape and the current signal on the layer thickness, the kinetic parameter, the pulse height, and the pulse duration for the thin diffusion model was examined. As a model, the (hemi)spherical electrode (unmodified and modified with a conductive porous layer) was chosen, as it considers a simplified model due to a unique distance to the electrode surface. By decreasing the layer thickness, smaller current peaks, a shift in the peak potential towards the standard potential, and a decrease in the half-peak width were observed. This observation was interpreted as an increase in the HET kinetic. Indeed, the same effects were obtained for the semi-infinite model with an increasing heterogeneous rate constant. In the porous structure, however, all these observations were attributed to the presence and depletion of electroactive species in the porous layer. Accordingly, the experimental results for porous electrodes can be (mis)interpreted. In addition, two other phenomena were observed for thin-layer regimes: (a) a ‘quasireversible maximum’ as a consequence of a deviation of the current response at long pulse times, which is due to depletion in the thin-layer finite diffusion space and (b) splitting of the current signal, for fast kinetic upon large amplitudes. As will be emphasized later, these observations can be used to diagnose or determine the contribution of thin-layer diffusion to the overall signal.

### 5.1. Cylindrical Electrodes and Arrays

For the illustration of the thin-layer diffusion in the mixed transport regime, in the above reviewed articles, previously reported simulations of the cyclic voltammetry and chronoamperometric response for various geometry and electrical properties of the porous layers were used. These simulations include variation in the shapes/geometry (cylinders (pillar), cylindrical pores, and spheres) of the porous layers, their dimensions (depth/high, surface area, and separation between shapes), and their electrical properties (active (e.g., conductive), inactive (e.g., non-conductive), or partially active)) in conjunction with a conductive or non-conductive substrate (electrode). Different combinations of the above parameters were used in the simulation of the (cyclic) voltammetric and chronoamperometric response. For example, as a model for the simulation of the irreversible oxidation of nicotine onto MWCNT-modified basal plane pyrolytic graphite (MWCNT-BPPG) [2], they considered vertically oriented cylindrical (pillar) electrodes on the non-conductive support [73]. In the latter, they modeled chronoamperometric response and the steady-state concentration profile for various substrate conductivities and cylinders (full, top-only, and side-only) and various cylinder radii and heights (depth), as well as the chronoamperometric response for the arrays of the above-mentioned cylinders. For the insulated cylinder arrays at a small depth, they obtained a transition from the planar Cottrellian response at short times, along the non-planar diffusion effects caused by the situation, where the diffusion layers of the neighboring cylinders are separated, to the longer times in which planar diffusion is again achieved due to the overlapping of the diffusion layers of the neighboring cylinders, as the diffusion layer exceeds the top of the cylinders. The latest was not observed for higher cylinders in the time frame of the simulation. For the top-only conductive cylinder, non-planar diffusion is quickly reached, and it is followed by strong dependence of the depth and radius of the cylinders, as for shorter cylinders, the final planar diffusion is more quickly attained. For fully conductive cylinders, at short times, a greater current can be expected due to an increase in the electroactive area. This is more evident at a higher depth of the cylinders; however, planar Cottrellian response cannot be achieved at longer times for deeper cylinders.

The same model of cylindrical arrays was used to study the difference in the current signal between staircase and analog voltammetry in the case of a porous electrode [74]. They found that, in the case of staircase voltammetry and for reversible kinetics, the signal of the voltammogram depends on the point of the current measurements, as the peak current decreases with an increase in the time when the current is measured. This has a large implication when measurements were performed at the different scan rates for the staircase voltammetry measurements.

Besides the above simulation model, other authors have designed, fabricated, and characterized (theoretically and experimentally) a gold-based micropillar electrode. Sanchez et al. [75] discovered many similarities in the behavior between porous and micropillar electrodes, especially for dense micropillars, and concluded that hemispherical diffusion plays a minor role in mass transport. Thus, planar diffusion is a process that controls overall mass. The current response is also governed by planar diffusion at slow scan rates in cyclic voltammetry (CV) and at longer times in chronoamperometry (CA), as the depletion of electroactive species between micropillars becomes complete, and planar diffusion from the bulk to the electrode surfaces dominates. Interestingly, the authors found that certain phenomena, such as hindered degassing and issues with the full penetration of the electrolyte, can occur in high-density micropillars. This leads to the conclusion that conditions within dense porous structures can differ significantly from those in the bulk solution.

Chen et al. [76] used polydimethylsiloxane (PDMS) micropillars sputtered with gold in order to obtain conductive layers. The resulting arrays were modified with a Pd-MWCNT nanocomposite to obtain a layer sensitive to sarcosine. However, the authors did not comment whether any mass transfer phenomena could potentially occur in this system.

### 5.2. More Complex Cylindrical Arrays

**Figure 1:**
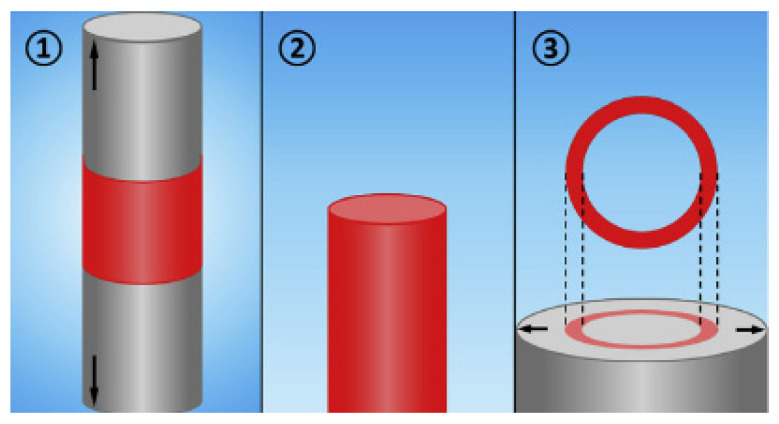
Illustrations of the models investigated: ① the ‘annular band’ electrode model, ② the cylinder electrode model, and ③ the ‘embedded ring’ electrode model. The black arrows in the figures represent infinitely expanding insulating areas [77]. Reproduced with permission from J. Electroanal. Chem.; published by Elsevier, 2019.

In further theoretical investigations, the Compton group introduced a new diffusion indicator to characterize the nature of the diffusion (linear vs. convergent) in chronoamperometric measurements. It is derived from simulations of more complex cylinder-shaped geometries, which, in addition to the previously mentioned variations in the cylinder, included the ‘annular band’ and ‘embedded ring’ models (Figure 1) [77].

They determined that the geometrical parameters under which diffuse fluxes toward micropillar electrodes, as defined by Aoki et al. [78] and Szabo et al. [79], can be used to predict the chronoamperometric response for these types of electrodes.

**Figure 2:**
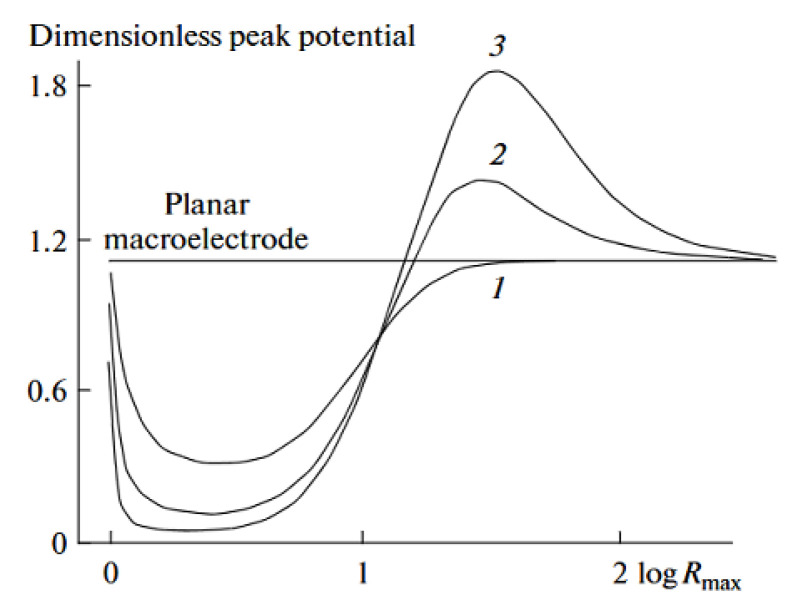
Plot of peak potential against *R*_max_ for a reversible system. Data are plotted for different values of film thickness with the peak potential for a planar macroelectrode plotted for comparison. Film thickness: (1) 30, (2) 100, and (3) 300 [80]. Reproduced with permission from Russ. J. Electrochem.; published by Springer Nature, 2012.

Finally, the cylindrical model was used for the prediction of the splitting voltammetric signal into double peaks for the porous film electrode, where electrolysis occurs at the substrate, at the porous film surface, and within it [80]. The simulation of the porous film used in this model includes the influence of the cylinder separation and cylinder depth (film thickness) on the current and current peak, using linear sweep voltammetry. For reversible kinetics, under boundary conditions (very high and very low surface density of the cylinders), a planar Cottrell response is obtained, which corresponds to the Randles–Sevcik limit. In the moderate surface density of the cylinders (moderate separation), the current increases, due to the increased electroactive surface area, followed by a decrease in the current and by a further decrease in the cylinder density. This decrease is explained by the fast depletion of the electroactive substance in the thin layer (in the pores), suggesting that not only a large electroactive surface area is important but also an amount of the electroactive specie in the pores. For a very high density of the cylinders, the electrode behavior can be depicted by the Randles–Sevcik limit (for the planar Cottrellian response), since the electrode acts as a planar electrode (where the electrode surface is the top of the cylinders). This behavior becomes more pronounced with the increasing cylinder depth, due to the increased electroactive surface area as well as the pore volume (increased amount of electroactive species in the pores). Accordingly, the shift in the peak current potential towards a higher overpotential than for the planar macroelectrode is expected with an increase in the separation between cylinders as a consequence of the increased thin-layer diffusion. This indicates that mass transport is not a limiting factor, as is the case with the planar macroelectrode. On the contrary, by increasing the cylinder density, a lower overpotential is expected, as the volume of the pores decreases. Accordingly, thin-layer diffusion is observed to a lesser extent due to the reduced amount of electroactive species in the pores and the shorter diffusion path between cylinders. In moderate cylinder surface density, a transition is observed from a decrease in peak current potential with decreasing cylinder density to an increase in the peak current at a high cylinder density. This transition can be explained in the same manner as described earlier (see current dependence at high cylinder density). Of course, this behavior becomes more pronounced with an increase in the cylinder depth. In the case of irreversible kinetics, a similar pattern of current peak as in reversible kinetics can be expected. However, at a high cylinder density, the peak current can split into two peaks. One is a consequence of the thin-layer effect, while the other is related to planar diffusion. This splitting depends strongly on the cylinder density, especially in the low current density region, as the current peak from thin-layer diffusion becomes more prominent with an increase in pore size, until the second peak, which corresponds to planar diffusion, is hindered. Interestingly, this separation cannot be observed for reversible kinetics, as the difference in the peak potential (for a given time frame of the experiment, e.g., a scan rate of 10 mV s^−1^) for thin-layer and planar diffusion, for such fast kinetics, cannot be observed (Figure 2).

### 5.3. Cylindrical Pores

**Figure 3:**
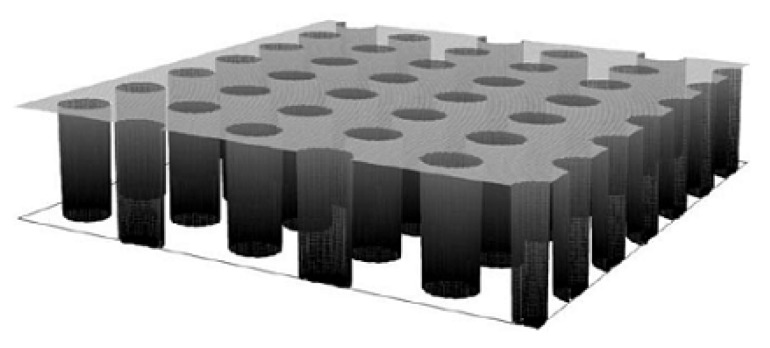
Model of the electrode surface [81]. Reproduced with permission from Electroanalysis; published by WILEY Analytical Science, 2008.

**Figure 4:**
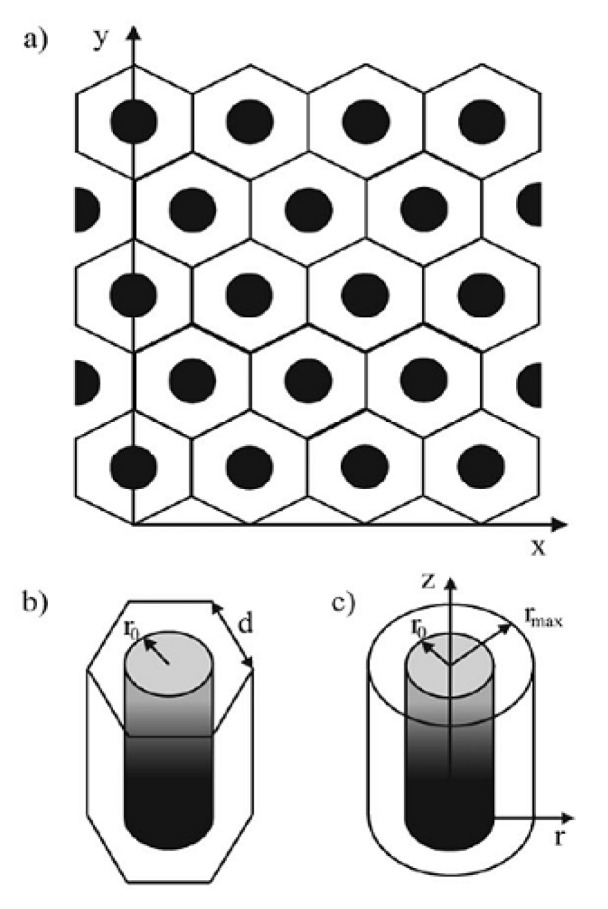
(**a**) Schematic diagram of the unit cell for an array of cylindrical pores; (**b**) single unit cell in Cartesian coordinates; (**c**) equivalent diffusion domain in cylindrical coordinates [81]. Reproduced with permission from Electroanalysis; published by WILEY Analytical Science, 2008.

Moreover, the same group has considered another geometry for the porous layer’s pores with cylindrical symmetry, known as the pinhole model [81,82] (Figure 3). To simplify the diffusion problem for this geometry, the diffusion domain was treated as cylindrical (with the pore as the axis of symmetry), and for the calculations, it was approximated as hexagonal with the same surface area to ensure that the entire domain was included ‘without empty space’ (Figure 4).

**Figure 5:**
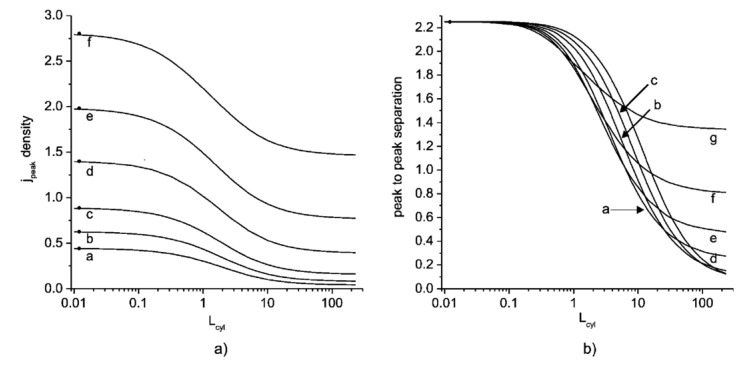
Peak current (**a**) and peak-to-peak separation (**b**) at different scan rates and cylinder depth values *L*_cyl_; infinitely fast rate of electrode transfer; (a) *σ* = 0.1, (b) *σ* = 0.2, (c) *σ* = 0.4, (d) *σ* = 1, (e) *σ* = 2, (f) *σ* = 4, (g) *σ* = 10. Dots show the values calculated for the flat macroelectrode limit with DigiSim [81]. Reproduced with permission from Electroanalysis; published by WILEY Analytical Science, 2008.

Simulations of the cyclic voltammetric response were performed at different pore depths, scan rates, and constants of heterogeneous electron transfer. It must be emphasized that the pores are considered electroinactive. Accordingly, the electrochemical reaction takes place on the underlying electrode surface. Regardless of the kinetics and the scan rate, the simulated voltammograms show a tendency to be similar to the voltammograms of the flat electrode (without pores) as the depth of the pores decreases. However, the current decreases with increasing pore depth at a constant scan rate. This was attributed to the hampered mass transfer from the bulk solution into the pores, which is obviously more pronounced at faster scan rates. Furthermore, this effect is more emphasized at faster scan rates, as the diffusion layer becomes thinner with an increase in the scan rate. Consequently, diffusion within the pores and diffusion from the bulk do not interfere with each other. This behavior has an influence on the peak-to-peak separation, since it is dependent on the mass transfer (with the same constant of the HET). Accordingly, the peak-to-peak separation becomes constant with an increase in the scan rate, as the electroactive species in the pores becomes depleted. Finally, this phenomenon is more pronounced in deeper pores (Figure 5).

This approach is successfully used to arbitrarily distinguish mass transport through a film (e.g., through a polymer membrane) from pore diffusion [83]. Depending on the various parameters, such as scan rate, pore depth, and diffusion coefficient, different types of simulated cyclic voltammograms were obtained, as follows, from steady-state (at slow scan rates), across Randles–Sevcik dependence, toward a thin-layer type of voltammograms for fast scan rates. Other parameters were also simulated for these two cases (film and pore), such as peak-to-peak separation, peak current, etc.

**Figure 6:**
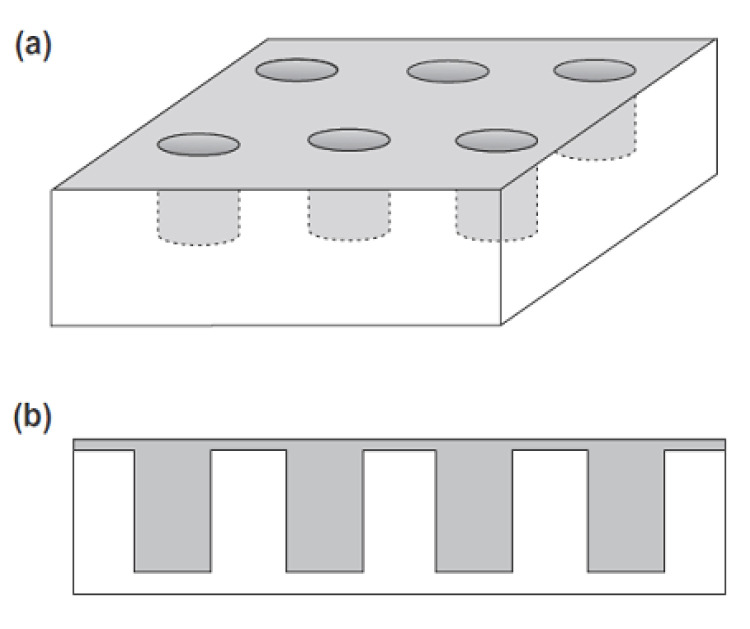
(**a**) Schematic of a porous surface; (**b**) side on view [84]. Reproduced with permission from J. Electroanal. Chem.; published by Elsevier, 2014.

The behavior of the voltammetric signal versus the geometrical parameters of the conductive cylindrical pores (support is insulating), together with the determination of the extent of this effect, was considered by Ward et al. [84]. The simulations were performed for the different scan rates and for fully reversible and fully irreversible kinetics for the various pore radius, depths, and separation between pores (Figure 6).

For fully reversible kinetics at fast scan rates, the voltammetric signal is expected to resemble those observed in the thin layer. This phenomenon depends on the cylinder pore depth, as increasing the depth allows thin-layer diffusion to be observed even at slower scan rates. This is followed by the dependence of the current peak upon the Randles–Sevcik equation. Of course, there is a transition region in which the contributions of the two diffusions predominate. For irreversible kinetics (slow kinetics), there is a threshold value for the pore depth at which the voltammetric signal shows an ‘apparent catalytic effect’. However, this model is not so important for the subject of this work, as it assumes that support is non-conductive.

**Figure 7:**
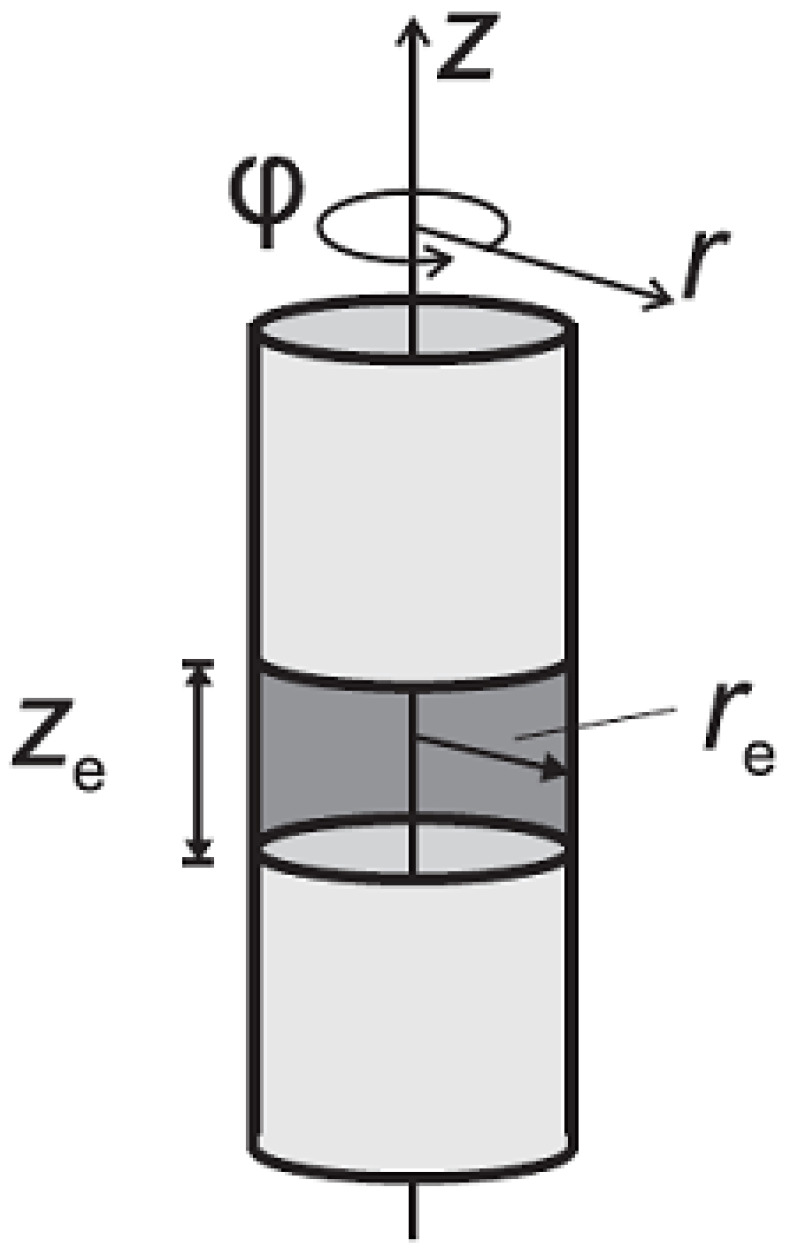
Band of conducting electrode of height *z_e_*, embedded in a hollow insulating cylinder of radius *r_e_* and the (*r*, *z*, φ) cylindrical polar coordinate system. The front section of the conducting band is drawn as transparent [85]. Reproduced with permission from J. Electroanal. Chem.; published by Elsevier, 2013.

The variation in the cylindrical pores was also considered for an infinite cylindrical tube with a partially electroactive inner surface (like a ring electrode), where the electroactive species are located in the cylinder interior surface (Figure 7) [85]. The variation in the height of the inner electroactive ring surface and the radius of the ring were investigated when changing the scan rate. The authors established four limiting cases. For an infinitely large cylinder radius, voltammetry governed by the Randles–Sevcik equation can be expected. As the radius of the cylinder decreases, thin-layer behavior occurs, because the length of the active area increases.

Finally, for the situation in which the radius and length converge to 0 (zero), the current is equivalent to that of a macroelectrode with an electroactive area twice the top surface area (hole) of the cylinder.

### 5.4. Spheres

**Figure 8:**
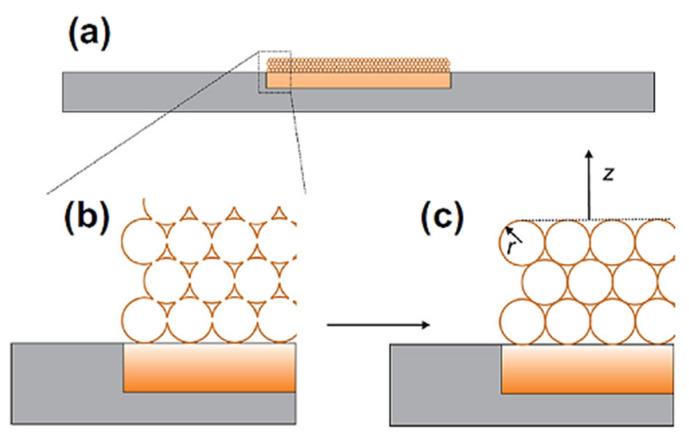
(**a**) Schematic cross-section of a porous electrode consisting of a series of hollow spheres supported on a disc electrode (the size of the sphere relative to the disc electrode is greatly exaggerated); (**b**) shows a zoomed in section, highlighting then interconnected nature of the spheres; and (**c**) shows a depiction of the model used to simulate electrochemistry at the electrode [86]. Reproduced with permission from J. Electroanal. Chem.; published by Elsevier, 2014.

The same group has developed a theory for the porous electrode based on the hollow conductive spheres (Figure 8) [86].

Furthermore, they were guided by the assumption that, in the case of the similar potentials of two analytes, the thin-layer effect can be used to separate these two current signals, while simultaneously lowering the overpotential. For the purpose of the experiment, diffusion between the spheres and inside spheres and the bulk was neglected. This allows the assumption that the reaction can take place in the spheres, at the electrode (substrate) surface, and on the top layer of the spheres. In the case of a small sphere radius, the overall electrochemical behavior is, therefore, similar to that of the flat electrode. Accordingly, the authors try to separate these models using mass transport. This model fits the case of a porous electrode fabricated using the drop-casting method with carbon nanotubes. For the flat electrode (diffusion from the bulk to the surface of the spheres), the authors relied on the previous considerations on the electrochemical reaction at flat heterogeneous electrodes [87,88], partially blocked flat electrodes [89,90,91], and flat microelectrodes with random or regular arrays [92,93]. Accordingly, they used the Shoup–Szabo equation for the chronoamperometric simulations, while for cyclic voltammetry, they relied on Butler–Volmer kinetics. In the case of chronoamperometry within a sphere, they reduced the spherical (symmetric) space to a one-dimensional system, which allows them to express the dependence of the concentration of the electroactive species according to Fick’s second law. This model is only modified in terms of the surface boundary condition, where Butler–Volmer kinetics is used.

**Figure 9:**
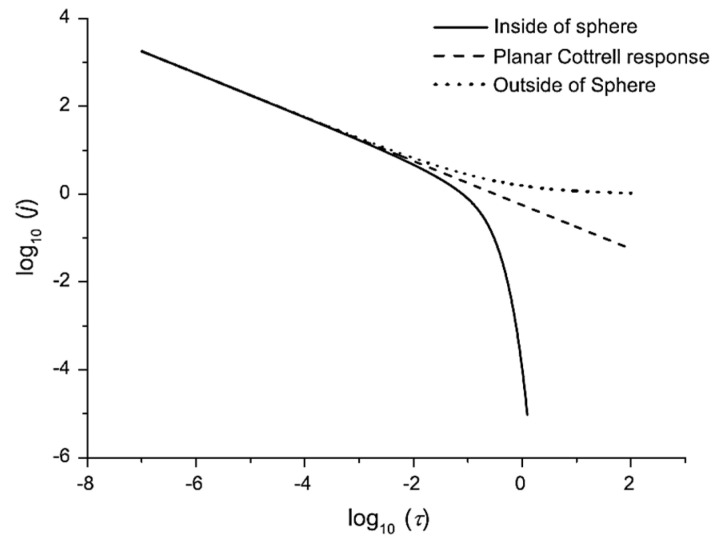
Chronoamperometric responses (in dimensionless parameters) of the inside of a sphere (solid line), a macrodisc (dashed line), and the outside of a sphere (dotted line) [86]. Reproduced with permission from J. Electroanal. Chem.; published by Elsevier, 2014.

**Figure 10:**
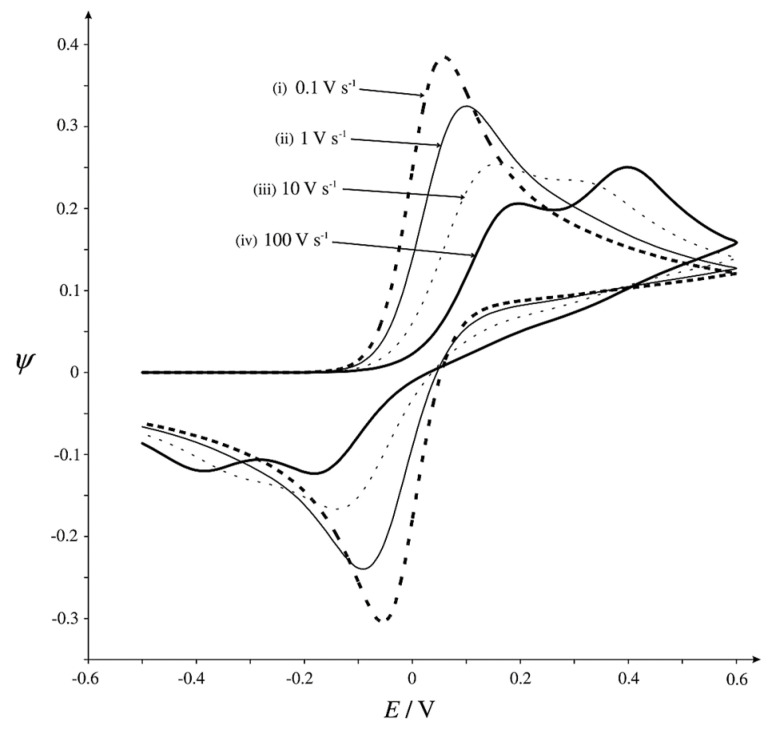
Simulated dimensionless current cyclic voltammograms for the *R*_0_ = 10 μm diffusion domain where the scan rate is (i) 0.1 V s^−1^, (ii) 1 V s^−1^, (iii) 10 V s^−1^, and (iv) 100 V s^−1^ [87]. Reproduced with permission from J. Electroanal. Chem.; published by Elsevier, 2004.

Simulated chronoamperometric data show that, at short times, there is no difference between the current generated inside the sphere and the flat electrode. The response follows the Cottrell equation, as the diffusion layer is close to the electroactive surfaces. However, at longer electrolysis times, the current within the sphere decreases rapidly, indicating a depletion of the electroactive species in the sphere’s volume (Figure 9). In contrast, at the same times (log *t*), the current (log *i*) of the flat electrode maintains a linear dependence, consistent with the Cottrell equation. This allows us, by simply adding the current response within the sphere (with the number of spheres known), including the parameter for the ratio of the flat electrode radius to the sphere radius, to approximate the total response of the porous electrode. Accordingly, the total chronoamperometric response at short times suggests a significant contribution of the thin-layer phenomenon when simulations are performed with a constant sphere radius. As expected, by increasing the number of spheres, the thin-layer response becomes more pronounced. Additionally, the thin-layer effect diminishes (within the time frame of the simulation) as the sphere radius decreases. Simulation data for cyclic voltammetry within the sphere reveal a strong dependence of the current on the scan rates. Since the entire electroactive substance can be consumed inside the sphere at low scan rates, a thin-layer response is observed, with the slope of the log υ vs. log *i* plot equal to 1, the same as for adsorption-controlled electrolysis. At fast scan rates, this depletion is not complete. Bearing in mind that at fast scan rates the diffusion layer is not as extended as at slow scan rates, diffusion occurs within the sphere. This has implications for the phenomenon of splitting the voltammetric signal (Figure 10), in the same manner as presented in previous work [80,86].

Generally, this splitting cannot be observed at fast scan rates (regardless of the kinetics) nor in systems with a large HET constant (reversible reactions?). This phenomenon is more prominent at slow kinetics; as the number of spheres increases, the radius of the spheres increases, while it is almost independent upon the surface of the flat electrode. The position of the peak potential is also the result of the interplay of electrochemical and mass transfer phenomena that occur both on the flat electrode surface and within the sphere at different electrochemical parameters. At fast scan rates, regardless of kinetics, splitting of the peaks cannot be noticed. At slow scan rates and fast kinetics, this splitting is less pronounced. The large splitting of the current peaks can be observed at slow scan rates and slow kinetics.

Elliot et al. [94] used the aforementioned diffusion indicator [77] to characterize the nature (convergent or linear) of the diffusion fluxes, qualitatively and quantitatively, toward a truncated electroactive sphere. By modeling ‘the one sphere’, they examined the different contributions, ranging from thin-layer to linear and convergent diffusion, depending on the extent of truncation of the sphere.

**Figure 11:**
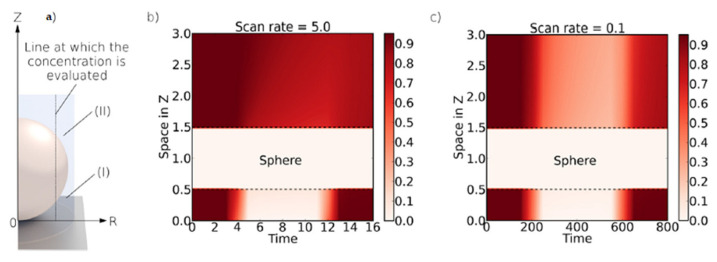
Concentration profiles as a function of Z and time evaluated for two different dimensionless scan rates and one layer of spheres. (**a**) Schematic depiction of the line at which the concentration profile is calculated. (**b**,**c**) Concentration profiles calculated for scan rates of 5 and 0.1, respectively. Please note that, for the clarity of presentation, voltammograms are modelled within a dimensionless potential range of ±10 rather than ±20 as in all other simulations [30]. Reproduced with permission from Appl. Mater. Today.; published by Elsevier, 2019.

Also, simulation models were used in unscrambling the ‘catalysis effect’ on an electrode modified with electroinactive spheres, which were considered a special case of the so-called ‘blocked electrode’ [89,90,91] in the works of Kätelhön et al. [30] and Yang et al. [95]. As these particles have a crucial impact, through mass transport phenomena, on the electrode electrochemical behavior, this can be easily misinterpreted as electrocatalysis. Of course, phenomena of voltammetric signal splitting can also occur, as previously reported [80,86,87]. Although unexpected and unusual behavior was described in previous reports [80,86,96,97], Kätelhön et al. [30] provided a description of the phenomena that led to the unusual variation in the peak-to-peak separation with the scan rate, as well as the decrease in the peak height with the increasing scan rate. Their explanation is based on the extent of the decoupling of mass transfer (diffusion) in different volumes. One volume is between the conductive electrode and the inactive sphere, while the other is the bulk (Figure 11).

They provide simulations of CV features, such as peak heights, peak position, and peak-to-peak separation that are affected by the thickness of the inactive layer, the size of the sphere, and the scan rate. The simulations reveal that, under the limiting conditions (extremely high or low scan rates), one can expect a similar peak-to-peak separation as for the bare electrode. This can be explained by classical voltammetry theory, as at fast scan rates, the diffusion layer does not spread from the electrode surface into the porous layer (due to the time frame of the experiment), which consequently has a similar diffusion profile to the bare electrode. At slow scan rates, there is sufficient time for diffusion from the bulk into the porous layer (and towards the electrode). Accordingly, the diffusion profile becomes similar to that of the bare electrode. Interestingly, substantial variation in the peak-to-peak difference in the intermediate region of the scan rates can be expected. This is a consequence of the interplay between the two aforementioned phenomena. At slightly higher scan rates, a strong decoupling of these two phenomena can be expected, leading to extensive depletion of the electroactive species in the volume between the sphere and the electrode. This is followed by low mass transfer from the bulk toward the electrode surface, resulting in a smaller peak-to-peak separation. At slightly slower scan rates, there is enough time for the diffusion of electroactive species from the bulk toward the electrode surface, which manifests as a higher peak-to-peak separation. Of course, before this diffusion occurs, the depletion of the electroactive species in the small volume of the electrolyte between the sphere and the electrode happens (thin-layer effect). This can be understood as the superposition (weak coupling) of these two processes. These phenomena are strongly dependent on the thickness of the layer. As the layer becomes thicker, less contribution from diffusion in the bulk solution is possible. As a result, at a certain scan rate, the peak-to-peak separation is lesser for a thicker layer. However, diffusion from the bulk affects the signal at slower scan rates (the diffusion path within the pore is longer for a thicker layer). Therefore, the maximum peak-to-peak separation can be expected at slower scan rates as the layer becomes thicker. Additionally, a reduced peak height at intermediate scan rates can be observed for the modified electrode, as a consequence of harnessed mass transfer toward the electrode. This effect becomes more pronounced as the layer thickness increases.

**Figure 12:**
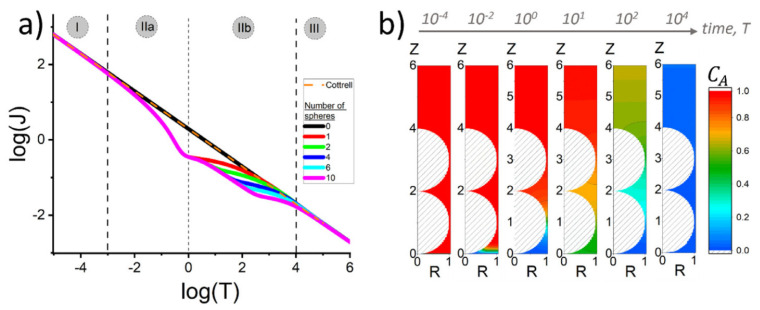
(**a**) Dimensionless flux-time transient to a planar electrode with n layers of surface blocks plotted in log-log form. At *T* = 0, a potential step is applied to the electrode from a potential of zero Faradaic current to a potential of diffusion-limited current to drive the reaction *A + e*^−^ → *B*. The logarithm is base 10. (**b**) Concentration profiles in the case of two layers of insulating sphere blocks (n = 2) modified on the electrode at different times. Other simulation parameters: *R*_max_ = 1.05, Δ*X* = 1 × 10^−12^, ΔT = 1 × 10^−12^, *ω*_X,T_ = 1.02, and *ε* = 500 [95]. Reproduced with permission from Appl. Mater. Today.; published by Elsevier, 2021.

Based on the previously mentioned model, Yang et al. [95] attempted to resolve the intertwined behavior of thin-layer and semi-infinite diffusion by simulating the chronoamperometric response for different thicknesses (number of layers) of the inactive sphere layers. For this purpose, they introduced the previously mentioned diffusion indicator [77,94] to separate the contributions and predict the occurrence of semi-infinite diffusion, the thin-layer effect, as well as the ‘bottlenecking’ phenomenon. First, the authors made a distinction between this model (‘blocked’ electrode) and diffusion within a film, as these two cases cannot be considered in the same way due to the fact that diffusion through a film is homogeneous with a reduced (but uniform) diffusion coefficient. In short, the authors divide the chronoamperometric response (e.g., mass transport within a porous structure) into different regimes that occur at different times (Figure 12). The first regime, at short times, is characterized by the chronoamperometric response agreeing with the Cottrellian behavior for the bare electrode. The second regime can be observed after a normalized time of 0.1 and shows identical behavior (deviation from the Cottrellian behavior, as flux drops more rapidly), regardless of the number of sphere layers. Accordingly, the phenomenon occurring in this regime can be a consequence of the perturbation of the concentration of the electroactive species in the first layer. The third regime occurs for electrodes containing two or more layers, where the concentration profile of the electroactive species exhibits an oscillatory character depending on the number of layers. These oscillations indicate the presence of ‘bottlenecks’ (higher concentration differences in the electroactive species, e.g., highest local flux) in the diffusion process, which occur where the gap between the layers is smallest, at the equator of the sphere. Finally, the fourth regime occurs at longer times and is again characterized by Cottrellian dependence. After this, the author explored the possibility of using the diffusion indicator to infer information observed by chronoamperometric measurements. Analysis by applying diffusion indicator reveals that, in the second regime, ‘thin-layer’ diffusion occurs to the same extent, regardless of the number of layers. So, this is a phenomenon that occurs within the first layer. At the border of the second and third regimes, the influence of diffusion from the above ‘compartment’ (the volume between the first and second layer of the sphere, or in the case of one layer, from the bulk) can be noticed. As diffusion toward the electrode is hampered by the funnels created by the spheres, steady-state behavior can be noticed. This can be recognized by a dramatic increase in the diffusion indicator toward positive values. This behavior occurs only twice, regardless of the number of layers, as these funnels, which made serial connection (one above the other), hamper the delivery of the electroactive species into the compartment between the first and second layers, making the maintenance of steady-state diffusion toward the electrode impossible. Finally, as it is an intuitive conclusion, this behavior is more emphasized, as the radius of the sphere is smaller.

All the above-mentioned theoretical and experimental approaches (summarized in Table 1) are based on mass transport phenomena and can be useful in the interpretation of the electrochemical phenomena that take place in various modified plane electrodes, as they consider the various geometry of the porous layers, electrical properties (inactive or active), or even partially conductive layers. It is clear that voltammetric and chronoamperometric responses can be (and usually are) an ensemble of different phenomena, including thin-layer diffusion, semi-infinite diffusion, and even adsorption phenomena, along with the possibility of an electrocatalytic effect, in cases where the porous layer is conductive. Therefore, the main effort should be directed towards separating these phenomena and detecting them.

## 6. Single-Entity Measurements

Recently, the Compton group published a series of articles related to the determination of the electrocatalytic behavior of the MWCNTs toward different species, such as bromine/bromide redox pair [29], oxygen reduction [98], the VO^2+^/VO^2+^ redox pair [99], nitrogen-doped MWCNTs for oxygen reduction [100], as well as studies of the electro-oxidation of the MWCNTs and amino-functionalized MWCNTs at different pH values [101]. The severity of these studies lies in the use of the single-entity approach to resolve the following question: are carbon nanotubes electrocatalytic or not?

**Figure 13:**
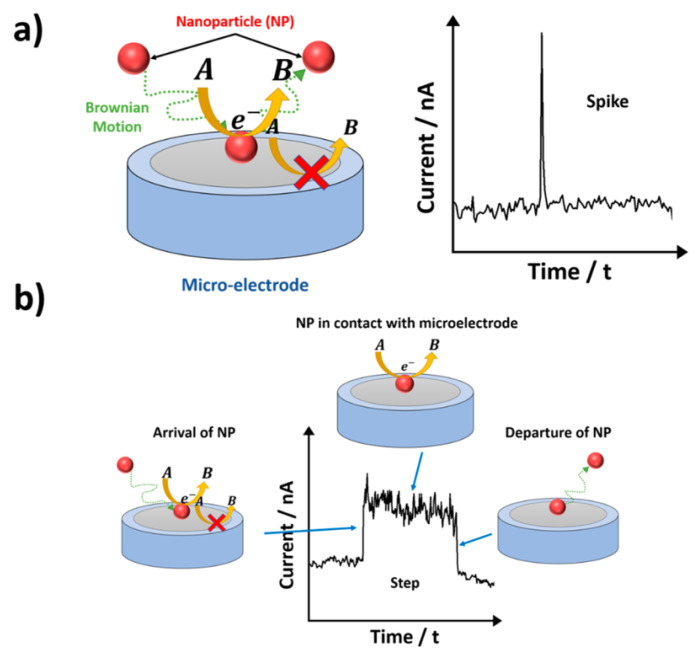
Schematic representation of (**a**) shorter duration impacts, resulting in current spikes and (**b**) a longer residence time at a microelectrode showing current steps. The arrows in yellow denote the mediated one-electron reduction reaction A(aq) + e^−^ → B(aq) [26]. Reproduced from J. Phys. Chem. Lett.; published by American Chemical Society, 2022. This publication is licensed under CC-BY 4.0.

A short review and perspective on the mentioned studies are presented in the work of Kumar et al. [26]. The single-entity approach is useful for exploring the electrocatalytic properties of various nanoparticles, including carbon nanotubes. This technique relies on the stochastic behavior (motion and ‘adsorption’) of the CNTs in their diluted solution toward (for motion) and onto (for ‘adsorption’) the electrode surface (Figure 13).

If the CNTs have electrocatalytic properties, then an increase in the current in the presence of the redox couple can be observed in the vicinity of the electrode or adhering to the electrode. By comparing the results obtained with drop-casted MWCNTs and those obtained using the single-entity approach with a pristine glassy carbon electrode, the authors concluded that the electrocatalytic effect of MWCNTs is present to some extent, as the rate constants for the oxidation and reduction in bromine and bromide are approximately one order of magnitude larger than those for the glassy carbon electrode [29]. However, the absolute value of the rate constants was around 10^−4^ and 10^−3^ for reduction and oxidation, respectively, which cannot explain the improved reversibility of the voltammetric responses of the drop-casted MWCNT electrode. Interestingly, for the VO^2+^/VO^2+^ redox pair [99], improved electron transfer was observed for the oxidation process, but it was retarded for the reduction process. This is not so surprising when considering the different transition states (e.g., changes in geometry, including breaking and forming chemical bonds) of these species. In addition, the redox reactions are complex in the sense of pH and proton transfer. Furthermore, a comparative study that included both the single-entity and drop-casted electrodes of the electrochemical reduction in the oxygen on MWCNTs was reported [98]. As the mechanism of this reduction involves a complex mechanism through quinone surface groups present on the MWCNTs, that issue is beyond the scope of this article. However, it is important to emphasize that, contrary to expectations, oxygen reduction occurs at a more anodic potential for the single-entity technique than for drop-casted MWCNTs. This behavior is explained by the irreversible destruction of the signal caused by the destruction of the quinone group on the MWCNTs when measurements were performed by CV. In contrast, due to the different settings of the single-entity measurements, fresh MWCNTs arrive onto the electrode surface, so the above-mentioned ‘poisoning’ is not present.

Finally, in a very interesting paper, Kumar et al. [102] demonstrated that using CV and single-entity measurements can be applied to distinguish thin-layer from adsorptive phenomena. They performed electrochemical oxidation of 4-hexylresorcinol on b-MWCNTs (bamboo-like MWCNTs) and revealed that distinguishing between thin-layer and adsorptive phenomena, even for different numbers of b-MWCNT layers, was inconclusive from the data obtained by voltammetry. The authors, based on calculations of the average charge per impact for different 4-hexylresorcinol concentrations, concluded that, if adsorption takes place on MWCNTs, then it must be of the multilayer (4–20 layers) kind of adsorption. When they applied an analysis for the calculation of the diffusion coefficient (*D*) of 4-hexylresorcinol, with the assumption of the diffusion-controlled process, they obtained a value of *D* in the order of magnitude of those reported in the literature. Thus, they concluded that voltammetric response is a consequence of thin-layer diffusion rather than an adsorption effect.

Besides the Compton group, other authors have also addressed the electrochemical behavior of the porous electrode. The effect of geometrical structure on the electrochemical behavior of carbon fiber, carbon nanospike, and carbon nanotube yarn electrodes was studied experimentally as well as through simulations by Cao et al. [103]. They demonstrated that an increase in geometry and scan rate results in more pronounced thin-layer behavior. In their simulation models, they successfully separated the CV signals into those belonging to diffusion and thin-layer electrochemistry, thus providing a comprehensive understanding of the influence of electrode geometry on electrochemical behavior.

To assess the role of CNTs in bioanalytical systems, Carrara et al. [4] studied three effects (Nernst, Cottrell, and Randles–Sevcik) using common electrochemical techniques. They demonstrated that, even in situations where CNTs apparently improve the analytical response, some effects, such as the Nernst effect, could be negligible or may even result in opposite trends in similar electrochemical systems. Also, they warned that many authors make the mistake of considering the specific active area as the sole reason for the improved signal. Finally, they concluded that various complex physicochemical processes govern the electrochemical response, and that to understand and enhance the performance of sensors based on CNTs (particularly those not related to H_2_O_2_ electrochemistry), much work still needs to be completed.

Based on fact that a tremendous number of synthesized compounds are used as electrocatalysists, Garcia et al. [104] contributed to establishing criteria for the characterization of electrochemical reactions occurring under finite diffusion conditions. By theoretical analysis, they presented that, in the case of non-highly efficient electrocatalysis, mass transport plays a crucial role. They also suggested that traditional methods for determining rate constants should be corrected by incorporating other modalities of diffusive fields and spatial domains.

Finally, it should be emphasized that mass transfer problems in various electrochemical systems continue to attract attention, as evidenced by a Special Issue devoted to this subject [105]. All the reviewed articles above demonstrate that alternative approaches can be used to distinguish the phenomena occurring within the porous layer structure, as well as the complexity and time frame of mass transport phenomena, thus posing a challenge to the electrochemical impedance spectroscopy (EIS).

## 7. Electrochemical Impedance Spectroscopy of the Porous Electrodes

For electrochemical devices, especially those designed for energy storage, porous electrodes dominate. Additionally, many porous materials have been widely used in electrochemical sensing devices. Since the processes occurring in porous electrodes can significantly differ from those at non-porous electrodes, one of the most powerful, non-invasive, and in situ tools for assessing and understanding phenomena in electrochemical systems is electrochemical impedance spectroscopy (EIS). These processes include electrode kinetics, capacitance effects, as well as mass transfer phenomena in solution and other specific processes. Due to the wide application of carbon nanomaterials (CNMs) in electrochemical and electrical systems, either as pristine materials or in composite form, a tremendous number of articles featuring EIS analysis can be found. These studies mostly focus on the development and characterization of microelectronics, electronic components, electroanalysis, energy conversion, energy harvesting, electrical energy storage, fuel cells, hydrogen storage, battery systems, electrochemical sensing, corrosion, composite materials, etc. Due to their porosity, CNTs are indispensable in such investigations. Many general information and data related to the analysis and interpretation of EIS spectra in these systems can be found in dedicated chapters of various scientific books or articles [106,107,108].

Historically, one of the earliest general works related to the application of EIS for the characterization of porous electrodes was reported by Candy et al. [109]. By comparing the simulated results with those of a Raney gold electrode, they concluded that the impedance technique can be used to evaluate the radius, depth, and number of cylindrical pores when no electrochemical reaction occurs at the electrode surface. Various approaches to the application of impedance methods for the investigation of porous electrodes were further developed by Raistrick et al. [110].

As two examples of the phenomena that take place in the porous structure, the authors related double-layer charging in the porous structure and fuel cell gas diffusion. Also, Lasia [111] simulated impedance spectra by the numerical addition of the impedances of pores and obtained an equation that described the potential and current distribution in cylindrical porous electrodes with semi-infinite and finite lengths. An important contribution to the understanding, interpretation, prediction, and deconvolution of diffusion-related EIS data, considering various pore geometries, was provided by Cooper et al. [112]. They found that impedance spectra can significantly deviate from the commonly used Warburg element in electrical equivalent circuits, potentially leading to the misinterpretation of impedance data. Furthermore, Tröltzsch and Kanoun [113] presented a general perspective on diffusion impedance in porous media using the transmission line model. They demonstrated how various processes—such as diffusion, charge transfer, charge accumulation, conductivity, and porosity—represented by different impedance models reported in scientific literature, are interconnected.

The effect of the pore size distribution on the transmission line model, as a new impedance model of porous materials in order to characterize the electric double-layer capacitor (EDLC), was developed by Song et al. [114]. In fact, they demonstrated the possibility of predicting the impedance of porous materials based on known pore size distributions. Similarly, Trishank Sharma [115] characterized the EIS response for various geometrical parameters of pores, such as radii, depth, density, and electrolyte conductivity. By describing the capacitive behavior of pores using a constant phase element, the authors concluded that these arrays can be represented as the sum of the EIS responses of individual pores. Also, a model for gaining insight into the physical processes, particularly the transient behavior of porous electrodes, was presented by Devan et al. [116]. Using their model, the authors successfully quantified various parameters, including ohmic resistance, charge transfer, and polarization resistance in dependence of various parameters, such as porosity, diffusion coefficient, particle size, etc. An enhanced model, based on the impedance transmission line with two transport channels, was presented by Bisquert [117]. He discussed the blocking of charge carriers at macroscopic boundaries at various frequencies and derived diagnostic criteria to determine whether the dispersion of the constant phase element is caused by the boundary or the inner surface.

As graphite is the most commercially significant anode material for Li-ion batteries, a tremendous number of articles involving EIS analysis of these devices can be found. Among the published articles, some provide a broader perspective on methods and the interpretation of porous electrode impedance spectra, such as those based on the transmission line model [118], or revisiting the physical interpretation of EIS spectra for graphite porous electrodes across different frequencies in the context of ion transport and pseudocapacitive behavior at high and middle frequencies [119]. In view of the extensive research on this topic, it remains a major challenge to compile a comprehensive overview.

Notably, Gruet et al. [120] investigated the EIS response of composite electrodes containing graphite (spherical particles) to assess and understand the degradation processes in Li-ion batteries. The authors presented a model that can predict optimal porosity in order to achieve the best performances upon the composition of the electrode.

One of the excellent reviews that provides an in-depth insight into the challenges, history, and journey of this subject is the review by Huang et al. [121]. One of the early efforts to yield data concerning the structure and composition of porous carbon materials, with an emphasis on the highly accurate determination of the available area through EIS, was carried out by Armstrong et al. [122].

Owing to the fact that carbon materials are widely used in energy storage, mostly in EDLC, Yoo et al. [123] attempted to establish an analytical method to correlate the EIS results with those obtained with the direct current method, such as galvanostatic charge–discharge cycling. They profiled rate capabilities of the porous carbon electrode in light of ionic accessibility and described the distribution of capacitance in respect to the latter. Suss et al. [124] presented both theoretical and experimental characterizations of hierarchical porous carbon aerogels, with varying structures of nanoscale pores integrated into a micron-scale porous network. The focus of the work presented by Abouelamaiem et al. [125] was on the development of a simple EEC model that can be used to describe porous carbon in supercapacitors and on the assessment of the role of pore morphology in improving the electrochemical performance of EDLCs. The authors concluded that, for optimizing porosity, pore size distribution, electrical pore resistance, and capacitance to achieve optimal charging and discharging capabilities, the assessment of relaxation times is crucial. Various inorganic oxide porous structures together with porous carbon were examined as materials for supercapacitors in the study by De et al. [126]. When compared to bulk materials, porous materials showed lower values of equivalent series resistance and charge transfer resistance. Also, the porous structure was found to act as an ion reservoir, stabilizing lattice expansion during cycling. Furthermore, the authors used simple Fick’s laws to calculate the diffusion coefficient in the examined porous materials.

Besides the general aspects of EIS analysis on porous structures or porous carbon, many carbon nanomaterials have been characterized by EIS, either purposefully or as part of studies related to specific subjects. Yang et al. [127] studied closed and open CNTs at different potentials to understand phenomena that can occur in lithium batteries, such as intercalation. Differences in morphology (e.g., open vs. closed) can significantly influence double-layer capacitance and diffusion phenomena. Closed nanotubes exhibited spectra that could be explained by adsorption phenomena, while open CNTs showed charge transfer from adsorbed intermediate species. Additionally, diffusion effects in open CNTs occur around both the inner core spaces and the outer surface, leading to faster diffusion processes. In contrast, diffusion in closed CNTs occurs only at the outer surface, resulting in slower diffusion. The authors also calculated the diffusion coefficient of Li ions in CNTs.

Leppänen et al. [128] conducted a comprehensive electrochemical and physicochemical analysis of various carbon nanomaterials, which included cyclic voltammetry and EIS. Their study demonstrated how electrochemical performance can be significantly influenced by small changes in the structural properties of the materials. Several electrochemical parameters were considered, including the point of zero charge, open circuit potential, double-layer capacitance, pseudocapacitance, working potential windows at various pH levels, reaction kinetics for both outer and inner sphere probes, and heterogeneous electron transfer. They found that small capacitance and a wide potential window are closely related to the reactivity of the nanomaterials, particularly for applications in electroanalysis. Interestingly, they applied a simple Randles model for their analysis, probably because they used an equimolar solution of Rh(NH₃)₃^2^⁺/^3^⁺, and there was no discussion of porosity (at least not for interpreting the EIS data).

The ultramicroelectrodes made of SWCNTs prepared by Dumitrescu et al. [129] showed very reproducible EIS spectra (even for electrodes with different diameters). These spectra were modeled using a simple EEC model and provided valuable data on the double layer capacitance, the diffusion coefficient of FcTMA^+/2+^, and the heterogeneous rate constant. The electrochemical response of twist-spun yarn MWCNTs (with a diameter of around 10 μm) at different potentials in both aqueous and non-aqueous solutions was studied by Mirfakhrai et al. [130]. They aimed to correlate the exponent of the constant phase element (CPE) with the physical structure of the yarn and found that the behavior of the CPE is closely related to the underlying physical and fractal structure of the yarns.

The electrochemical activity of a composite material consisting of SWCNTs and MWCNTs, along with silver, was investigated by Niessen et al. [131] to explain their response. They found that the EIS data were cross-correlated with those obtained from cyclic voltammetry (CV), revealing that more than 90% of the total charge was stored electrostatically (for commercially obtained CNTs). The study also raised doubts about whether CNTs can act as a hydrogen reservoir. Also, various composite materials containing mixtures of 2D and 3D carbon materials [132,133] were characterized using various electrochemical techniques, including EIS, to improve the efficiency of electrodes for high energy density supercapacitors.

Comparative EIS studies on SWCNTs and MWCNTs polymer nanocomposites to optimize the material composition for biosensor applications were presented by Tertis et al. [134]. They proposed a mechanism involving adsorption and relaxation processes. Additionally, Galicia et al. [135] utilized EIS to provide a perspective for characterizing CNT–chitosan scaffolds under flow conditions. They discovered that the transition from single to mixed diffusion and charge transfer mechanisms controls the overall response. As a conclusion, they suggested the potential to use chitosan–CNT composites for applications in flow conditions.

Recently, Han et al. [136] treated SWCNTs with O_2_ plasma to introduce oxygen-containing groups for enhanced sensitivity. Using EIS, the authors analyzed spray-coated CNTs on a platinum electrode. As the SWCNT layer thickness increased, they observed an increase in charge transfer resistance due to the reduced effective electrode area, resulting from decreased roughness and the porosity of the layer. In addition, the time constant was lowered due to the improved diffusion, as the charge transfer resistance decreased.

## 8. Conclusions

This review focuses on addressing the following fundamental question in carbon nanotube (CNT) electrochemistry: What is the role and purpose of incorporating CNTs into electrochemical sensors? Although CNTs are widely used in the development of electrochemical sensors, there is still a lack of clarity regarding the exact purpose of their involvement. To understand this, it is essential to establish the relationship between their physical and electrochemical properties and their influence on electroanalytical signals. Challenges arise from the blurred boundary between physical and electrochemical influences. In fact, the interplay, or even synergy, between physical and electrical properties may occur. This requires a meticulous approach to modeling CNT electrochemical systems, incorporating a variety of physical and electrical parameters (conductivity or non-conductivity of the basic ‘particle’ and/or substrate). These complex relationships can be studied using diverse electrochemical methods, from amperometry to voltammetry. Consequently, several theoretical models have been proposed to describe the phenomena occurring in CNTs during electrolysis.

Generally, these models, based on chronoamperometric and voltammetric methods, predict that the electrochemical response reflects mass transport processes, which can be described by a combination of semi-infinite and thin-layer diffusion. However, this mixed mass transport regime presents various manifestations, as it depends on the physical properties of the ‘basic particles’ (such as geometry, shape, orientation, and size, which serve as building blocks of the porous layer), electrical properties (e.g., conductivity), and electrochemical parameters (e.g., the kinetics of heterogeneous electron transfer and reversibility). The latter allows us to distinguish and estimate the electrochemical signal by adjusting the appropriate voltammetric parameters (e.g., scan rates) to obtain differences in the peak-to-peak separation, high peak currents, or even, splitting the voltammetric signals that are related to the different mass transport regime. The reliability of these findings is further improved when chronoamperometry is introduced, providing a diffusion indicator that offers both qualitative and quantitative insights into the nature of diffusion fluxes.

Although all the developed models can be used to elucidate the electrochemical phenomena occurring in CNTs, the simple question of whether carbon nanotubes act as electrocatalysts remains unanswered. Fortunately, single-entity experiments conducted on microelectrodes for selected electroactive species revealed that the reversibility of the electrochemical system cannot be explained by an improved heterogeneous electron transfer due to the presence of CNTs. It is important to note that this statement should be taken ‘with a grain of salt’, as it as it relevant only to specific cases (see reference [91]). Additionally, single-entity approaches can help differentiate between adsorption and thin-layer diffusion, as the electrochemical results for these two processes are nearly identical.

As presented, it is challenging to draw a definitive conclusion on this subject, as the various theoretical models need to be integrated into a unified theory. This would involve merging and simplifying the mathematical calculations, ultimately streamlining the approach. Additionally, a challenge arises from the fact that many reports on the use of CNTs for electroanalytical purposes do not focus on the role of CNTs in the architecture of the sensors. Moreover, the conclusions drawn from experimental results are often affected by the variability in the properties of CNTs, as they are sourced from different producers, and these properties can vary depending on the specific electroactive species involved. Other complications, such as adsorption within the CNT layer or the ‘fouling effect’, may result in a ‘blocked’ electrode. These are issues that future studies will need to address.

The approaches discussed above are designed as ‘alternatives’ and ‘challenges’ to electrochemical impedance spectroscopy (EIS), which is extensively used for analyzing and clarifying various electrochemical and physical processes in porous layers.

This review also provides a brief overview of the use of electrochemical impedance spectroscopy (EIS) in understanding mass transport phenomena in porous environments. Although this technique is highly effective for gaining insights into diffusion processes, its interpretation can be challenging. This is due to the necessity of using impedance theory and modeling, which require precise information about the system’s geometry, the defined interface between the electrode and electrolyte, and so on. Despite the large number of articles published on EIS and porous structures, limited information is available for clarifying and assessing mass transfer phenomena in carbon nanotubes (CNTs) when they are used in electrochemical sensors. This points to a future perspective on interpreting EIS data, in light of the insights gained through models, simulations, and experiments. Furthermore, the connection between cyclic voltammetry and EIS, as discussed in [137], is crucial for elucidating phenomena in the electrochemical system in real time and simultaneously.

## Figures and Tables

**Table 1:** Parameters, electrochemical methods, and analyte used in experimental and simulation studies.

Porous Layer	Conductivity of		Electrochemical Method(s) *		Analyte(s)	Reference
	Substrate	Porous Layer	Simulation	Experimental		
SWCNT	GC (conductive)	N/A	CV, LSV	CV	[Fe(CN)_6_]^4−^	[68]
MWCNT	GC (conductive)	N/A	CV	CV, CA	dopamine	[71]
MWCNT	BPPG (conductive)	N/A	CV	CV	nicotine	[2]
Porous	conductive	conductive	DPV, SWV, LSV	-	-	[1]
Cylindrical/micropillar	conductive or non-conductive	conductive, non-conductive, partially conductive	CA	-	-	[73]
	conductive	partially conductive	CV	-	-	[74]
	metallized SiO_2_ (conductive)	gold (conductive)	CA, CV	CA, CV	[Fe(CN)_6_]^4−^	[75]
	gold sputtered PDMS	drop-casted Pt–Pd/MWCNTs	-	CV, CA	sarccosine	[76]
	non-conductive	partially conductive	CA	-	-	[77]
	conductive	conductive	-	LSV	-	[80]
Cylindrical pores	conductive	non-conductive	-	CV	-	[81]
	conductive	non-conductive	-	CV	-	[83]
	non-conductive	conductive	-	LSV	-	[84]
	no substrate	partially conductive	-	LSV	-	[85]
Spheres	conductive	conductive spheres, reaction inside and on the spheres surface	-	CV	-	[86]
	no substrate	partially conductive spheres conductive, semi-spheres	-	CA	-	[94]
	conductive	non-conductive	-	CV	-	[30]
	conductive	non-conductive	-	CA	-	[95]

* CA—chronoamperometry; CV—cyclic voltammetry; LSV—linear sweep voltammetry; DPV—differential pulse voltammetry, SWV—square wave voltammetry.

## Data Availability

The data presented in this study are available within the content of this article.
